# Callose metabolism and the regulation of cell walls and plasmodesmata during plant mutualistic and pathogenic interactions

**DOI:** 10.1111/pce.14510

**Published:** 2022-12-19

**Authors:** Liam German, Richa Yeshvekar, Yoselin Benitez‐Alfonso

**Affiliations:** ^1^ Centre for Plant Sciences, School of Biology University of Leeds Leeds UK

**Keywords:** beta 1,3 glucanases, callose synthases, callose turnover, intercellular signaling, plant−microbe interactions, plasmodesmata proteins, symbiosis

## Abstract

Cell walls are essential for plant growth and development, providing support and protection from external environments. Callose is a glucan that accumulates in specialized cell wall microdomains including around intercellular pores called plasmodesmata. Despite representing a small percentage of the cell wall (~0.3% in the model plant *Arabidopsis thaliana*), callose accumulation regulates important biological processes such as phloem and pollen development, cell division, organ formation, responses to pathogenic invasion and to changes in nutrients and toxic metals in the soil. Callose accumulation modifies cell wall properties and restricts plasmodesmata aperture, affecting the transport of signaling proteins and RNA molecules that regulate plant developmental and environmental responses. Although the importance of callose, at and outside plasmodesmata cell walls, is widely recognized, the underlying mechanisms controlling changes in its synthesis and degradation are still unresolved. In this review, we explore the most recent literature addressing callose metabolism with a focus on the molecular factors affecting callose accumulation in response to mutualistic symbionts and pathogenic elicitors. We discuss commonalities in the signaling pathways, identify research gaps and highlight opportunities to target callose in the improvement of plant responses to beneficial versus pathogenic microbes.

## Brief summary statement

In this review, we expose our current understanding of the mechanisms that regulate callose accumulation in response to microbes. The aim is to identify research gaps and highlight opportunities to target callose in the improvement of plant responses to beneficial versus pathogenic microbes. Information was missing on the signaling pathways and interactions controlling the expression and activity of callose metabolic enzymes and their relative contribution to the response to microbial symbionts. Knowledge also lacks on the evolutionary and physicomechanical features that selected callose to function in this process. We also highlight opportunities for stronger plant defenses that emerge from priming callose using non‐pathogenic and mutualistic microbes.

## 1 INTRODUCTION

Plants interact with a variety of microorganisms during their lifetime establishing symbiotic associations. Some of these interactions lead to pathogenesis, whereas others mutually benefit the plant and the microbe. Mutualistic symbiosis occurs when plant roots interact with arbuscular mycorrhizal fungi and/or with soil‐borne nitrogen‐fixing bacteria, exemplified by the genus Rhizobium (recently reviewed by Lebedeva et al., 2021; Roy & Müller, 2022). Arbuscular mycorrhizal fungi facilitate the exchange of water and nutrients between plant roots and soil. Rhizobia fix nitrogen in legume roots by inducing the formation of specialized organs named nodules. On the other hand, pathogenesis is caused by a wide range of bacteria, viruses, fungi and other microorganisms that are detrimental to plant growth and lead to yield losses.

**Figure 1:**
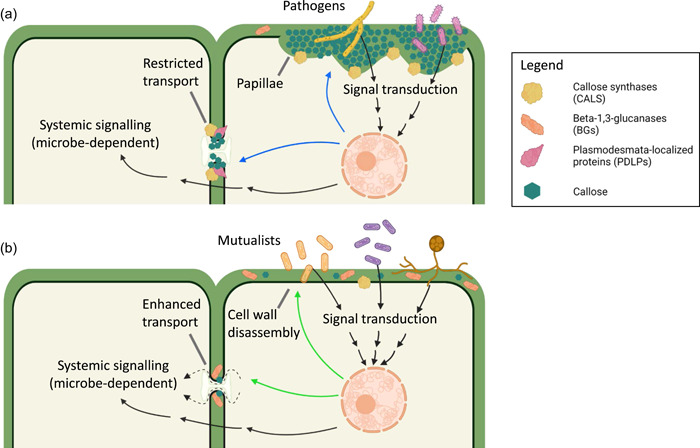
A simplified model of callose regulation in response to microbial interactions. (a) Pathogenic interactions (produced when plants are exposed to pathogenic bacteria and fungi) induce signaling pathways leading to changes in gene expression (black arrows). Among the responsive genes, there are callose synthases (CALS), plasmodesmata‐located proteins (such as PDLPs) and beta‐1,3‐glucanases (BGs) which modify plasmodesmata cell walls and participate in papillae formation to restrict pathogen invasion and symplasmic transport (blue arrows). Signals also travel apoplastically to neighboring tissues inducing systemic and non‐cell autonomous responses in a microbe‐dependent manner (black arrows across cell walls). (b) Plant mutualistic interactions (as established with mycorrhiza fungi, rhizobia and other endophytes) also trigger signaling pathways that target CALS and BG expression to modify callose turnover (synthesis vs. degradation) in cell walls. In the case of rhizobia, and perhaps other mutualists, activation of BGs functioning in cell wall disassembly and at plasmodesmata (green arrows) have been described. As a result, the transport of unknown symplasmic signals is enhanced (discontinuous arrows) which also contributes to systemic signaling. Figure 2 describes in more detail the effects of CALS and BG on plasmodesmata and Figure 3 highlights some of the signaling pathways involved in these responses. Created with BioRender.com.

To penetrate plant cells, both mutualistic and parasitic symbionts need to overcome the physical barriers imposed by the cell wall. Plant cell walls are made by a network of cellulose microfibrils connected by hemicelluloses, pectic polysaccharides and proteins (B. Zhang et al., 2021). The relative composition and architecture of this network determine cell wall mechanical properties, and plants have evolved to modulate these features to block pathogen penetration. Both pathogenic and mutualistic microbes deploy a set of cell wall digestive enzymes to facilitate their invasion (Hansen & Nielsen, 2018; X. Li et al., 2018; Malinovsky et al., 2014; A. Wang, 2021). To reinforce cell walls against fungal pathogens, plants synthesize callose, a beta‐1,3 glucan that interacts with cellulose‐forming papillae (Houston et al., 2016; Y. Wang et al., 2021) (Figure 1). Besides callose, the papillae contain antimicrobial chemicals such as phytoalexins, reactive oxygen species and defensins, which may act to eliminate invading parties. Callose also plays a role in controlling the spread of microbial infections by restricting the intercellular movement of proteins and effectors via the symplasmic route formed by plasmodesmata (Z. Li, et al., 2021 and references herein). Moreover, the degradation of callose at plasmodesmata regulates the formation of infection threads and nodules induced by rhizobia in *Medicago truncatula* roots (Gaudioso‐Pedraza et al., 2018).

**Figure 2:**
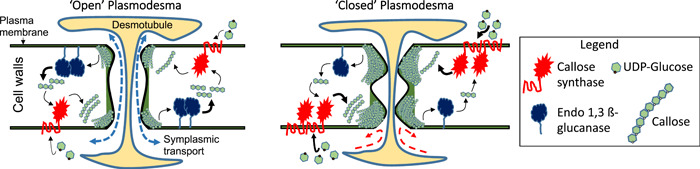
Callose mediated regulation of plasmodesma. A cartoon representing ‘open’ plasmodesma (left) and ‘closed’ plasmodesma (right) due to over‐accumulation of callose in cell walls. Plasmodesmata are intercellular channels inserted in cell walls and delimited by two membranous domains: a plasma membrane and a desmotubule connecting neighboring cells. The cytoplasmic sleeve between these two membranous domains is the path for symplasmic molecular transport (dotted arrow). Callose accumulation depends on cytoplasmic available UDP‐glucose and the concerted activity of callose synthases (CALS) and beta‐1,3‐glucanases (BG) colocalized at plasmodesmata. Callose determines plasmodesma cytoplasmic aperture restricting symplasmic transport. When callose degradation is favored (e.g., by up‐regulation of BGs showed by thicker arrows in the model on the left), symplasmic molecular transport is enhanced. When callose accumulates (e.g., higher CALS activity showed by thicker arrows in the model on the right) symplasmic transport is restricted (red discontinuous arrows). See also accompanied legend.

Plasmodesmata are intercellular pores traversing cell walls that mediate the local and systemic transport of molecular signals to coordinate developmental and environmental responses (Figure 2). Plasmodesmata are often visualized as concentric pores formed by an outer specialized membranous domain that is a continuation of the plasma membrane, and an inner desmotubule which connects the endoplasmic reticulum (ER) of neighboring cells (Cheval & Faulkner, 2018; Paniagua et al., 2021). Molecular transport occurs through the cytoplasmic sleeve, the space between the plasma membrane and the desmotubule. Callose deposited in the paramural space around the plasmodesmata form a collar that allegedly pushes the plasma membrane against the desmotubule to constrict the space available for transport (Fitzgibbon et al., 2010; Wu et al., 2018). The mechanical properties and interactions of callose with other cell wall polysaccharides modulate its biological function (Abou‐Saleh et al., 2018; Schneider et al., 2016). Callose accumulation correlates with a reduction in macromolecular trafficking, whereas callose degradation facilitates transport through plasmodesmata (Sager & Lee, 2014) (Figure 2). Other mechanisms, such as changes in plasmodesmata frequency or ultrastructure, are described to modify symplasmic transport allegedly independent of callose regulation. Various proteins which contribute directly or indirectly to callose metabolism and intercellular trafficking are found localized at plasmodesmata; thus, callose turnover is a dynamic process suited for rapid plasmodesmata regulation (Paniagua et al., 2021).

The role of callose in plant development and response to biotic and abiotic stresses has been studied; however, there is a significant lack of knowledge of the mechanisms that regulate callose synthesis/degradation in one process vs the other and the crosstalk between these pathways. In this review, we revisit current research on the mechanisms involved in callose metabolism at and outside plasmodesmata microdomains. We focus on plant symbiosis as an interesting system to evaluate the contrasting effects of modifying callose and highlight a few articles that implicate callose in establishing plant mutualistic symbiotic interactions. The aim is to reveal the intricate pathways plants use to regulate callose differentially and to identify opportunities in this area for future research.

## 2 ENZYMATIC SYNTHESIS AND DEGRADATION OF CALLOSE

Callose synthesis/degradation (i.e., turnover) is finely regulated at the cell wall by the activities of enzymes belonging to the callose synthase (CALS), also known as glucan synthase‐like (GSL) and β‐1,3 glucanase (BG) families (Figure 2). CALSs are classified as processive glycosyl transferases and catalyze the transfer of UDP‐glucose to form a β‐1,3 glucoside backbone (Lombard et al., 2014; Stone, 2006). CALSs are encoded by a multigene family in most plant species, with 12 reported genes in *Arabidopsis*, named CALS1‐12 or GSL1‐12. The genes encode proteins with multiple transmembrane domains, a UDP‐glucose catalytic site, and a glycosyltransferase domain. Different members function in different tissues or cellular domains or in response to different developmental and environmental cues. For example, CALS7/GSL7 plays a crucial role in the sieve plates and phloem development (Barratt et al., 2011), GSL8/CALS10 regulates cell plate formation during cell division and, together with CALS3/GSL12 regulate plasmodesmata connectivity (X.Y. Chen & Kim, 2009; Vatén et al., 2011).

Like the widely studied cellulose synthases, CALSs are part of a membrane complex that includes UDP‐glucose transferase, a GTP hydrolase, annexin and a sucrose synthase homolog (Schneider et al., 2016; Verma & Hong, 2001). CALSs are targeted to the plasma membrane through exocyst assembly that brings together vesicle‐associated subcomplexes. Particularly the exocyst family 70 is essential to target CALSs to the plasma membrane (He & Guo, 2009). In tobacco, gene silencing of the exocyst subunits led to reduced callose deposition in response to a bacterial pathogen (Du et al., 2018). Proteins involved in the regulation of vesicle trafficking, for instance, Rab (Rat sarcoma virus‐related in brain)‐ type GTP hydrolases (Rab‐ GTPase), also play a role in CALS membrane targeting (Cvrčková et al., 2012; Hála et al., 2008). Other proteins interact with the CALS/GSL complexes. For example, GSL6 was purified together with PHRAGMOPLASTIN, a dynamin‐like protein involved in cell plate formation (Hong et al., 2001). The identification of these interacting proteins evidence a mechanism for posttranslational regulation in CALS activity, which is also modified by changes in the protein phosphorylation status and proteolysis (see Schneider et al., 2016; and Wu et al., 2018).

The hydrolysis of callose is catalyzed by BG proteins encoded by members of the glycosyl hydrolases family 17 (Figure 2). These proteins contain a catalytic domain that degrades β‐1,3 glucosidic linkages and, in some cases, they harbor a carbohydrate‐binding module 43 that target callose (Leubner‐Metzger and Meins, 1999). Glycosyl hydrolases family 17 is a large multigenic family (50 genes identified in *Arabidopsis*) that can be grouped depending on phylogenetic relations, structural features and gene expression analysis (Doxey et al., 2007; Gaudioso‐Pedraza & Benitez‐Alfonso, 2014; Paniagua et al., 2021). Some members of this family are targeted to the apoplastic side of the plasma membrane via a C‐terminal glycosylphosphatidylinositol‐anchor, whereas others are secreted to the apoplast (Gaudioso‐Pedraza & Benitez‐Alfonso, 2014). Cleavage of the glycosylphosphatidylinositol‐anchor or modifications in membrane lipid composition, mediated for example by phospholipases, can modify the targeting, thus microdomain specific activity, of these enzymes (Grison et al., 2015; G.H. Lee et al., 2016). Differences in protein structure and intracellular localization are associated with diversity and/or redundancy in function. For example, the spreading of the Turnip vein clearing virus was restricted in an *Arabidopsis* mutant in a plasmodesmata‐localized BG protein (named *atbg_pap*) but not in mutants in an ER‐ and extracellularly localized stress responsive BG protein (named *atbg2*) (Zavaliev et al., 2013). Plasmodesmata‐located BGs coexist with another family of proteins (named plasmodesmata‐callose binding proteins or PDCBs) containing a callose‐binding domain exclusively. Ectopic expression of these PDCBs increases callose allegedly by creating complexes or structures that stabilize or protect the polysaccharide against degradation (Simpson et al., 2009).

The detailed mechanisms regulating CALS and BG activities have not been thoroughly dissected, but signaling pathways mediated by calcium, phytohormones and receptor‐like kinases are at the core. It has been reported that changes in cytoplasmic calcium levels contribute to callose deposition and plasmodesmata closure (Holdaway‐Clarke et al., 2000). In fact, previous research shows that most CALS activity in the plasma membrane depends on calcium (Him et al., 2001). This is likely due to the formation of complexes with annexins which are calcium‐dependent membrane‐binding proteins. In apparent contradiction, CALS9/GSL10 was found to induce ectopic callose accumulation in plants exposed to low calcium in the medium (Shikanai et al., 2020). The authors explained that differences between apoplastic and intracellular calcium levels might induce calcium influx, increasing cytoplasmic concentration, which in turn regulates CALS activity. In vitro CALS assays comparing the membrane‐enriched proteomic fraction and the plasmodesmata‐enriched fraction isolated from poplar cell cultures indicates the existence of calcium‐independent activities at the plasmodesmata (Leijon et al., 2018). This finding suggests that both calcium‐dependent and calcium‐independent CALS activities coexist at plasmodesmata, although their differential contributions to the regulation of symplasmic permeability are unknown.

Besides calcium, phytohormones such as auxins regulate callose synthesis and degradation (Band, 2021; Bharath et al., 2021; Kalachova et al., 2020). The AUXIN RESPONSE FACTOR7 positively regulates the expression of GSL8 in *Arabidopsis*, enhancing de novo callose synthesis at plasmodesmata and leading to reduced cell‐to‐cell permeability (Han et al., 2014). Increased callose decreases auxin fluxes leading to auxin accumulation, which in turn inhibits AUXIN RESPONSE FACTOR7 and GSL8 expression reducing callose levels at plasmodesmata. This feedback loop establishes the correct spatiotemporal position of auxin maxima that determines important biological processes such as the phototropic response or the outgrowth of lateral root primordia (Han et al., 2014; Sager et al., 2020). The regulation of callose metabolism at plasmodesmata in response to auxins (and other phytohormones) appears to be mediated by specific receptor‐like proteins named plasmodesmata‐localized proteins (PDLPs). PDLPs encode eight proteins containing domains of unknown function 26 with canonical disulphide bridges and transmembrane domains. PDLP overexpression leads to callose deposition, reduced intercellular transport and severe growth phenotypes (Thomas et al., 2008). Auxin‐induced expression of PDLP5 influences callose synthesis and plasmodesmata transport through direct or indirect interaction of PDLP5 with CALS1 and CALS8 (Cui & Lee, 2016). The mechanism behind PDLP5‐CALS regulation is not clear. Studies identified alanine and glycine residues spaced by three variable amino acids as a motif required for PDLP homomeric and heteromeric‐interactions (possibly with CALSs). Mutations in this motif compromise PDLP5 activity and capacity to regulate cell‐to‐cell transport (X. Wang et al., 2020).

As described for auxin, abscisic acid, gibberellic acid, salicylic acid and reactive oxygen species, among other signals, regulate the expression of CALS and BGs, controlling the transport of important developmental and stress‐responsive factors. Work identified an abscisic acid‐dependent mechanism involving callose in the seasonal control of bud dormancy in aspen trees (R.K. Singh et al., 2019; Tylewicz et al., 2018). Dormant buds exposed to short photoperiods accumulate high abscisic acid levels and high expression of CALS1, resulting in increased callose deposition and restricted expression and transport of growth factors. When conditions are favorable, gibberellic acid reverts this effect by inducing the expression of BGs to degrade plasmodesmata‐callose. This facilitates the transport of transcription factors such as flowering locus T that activate meristem development and outgrowth (Rinne et al., 2016).

To summarize, CALSs and BGs have been identified as the enzymes responsible for callose synthesis and degradation in cell walls. CALSs are part of a multimeric complex that regulate its activity and that includes, among others, proteins with calcium‐binding domains and dynamin‐like proteins. At plasmodesmata, subfamilies of membrane‐targeted BGs and CALSs have been identified, as well as other proteins that participate in callose regulation (i.e., PDCBs and PDLPs) either by recruiting and stabilizing callose or by physically interacting with the metabolic enzymes. Signaling molecules, including calcium, reactive oxygen species and phytohormones trigger signaling pathways that control the expression and/or activity of CALSs and BGs, modulating callose levels in cell walls. Some of these molecules (e.g., salicylic acid and reactive oxygen species) play essential roles in coordinating plant responses to microbes. Their role in callose regulation in the context of plant−microbial interactions will be discussed in more detail in the next section.

## 3 REGULATION OF CALLOSE METABOLISM AS A GENERAL RESPONSE TO MICROBIAL INTERACTIONS

Invasion by many pathogenic viruses, bacteria and fungi triggers alterations in callose levels in cell walls which can act as both: a defensive mechanical barrier against penetration and/or pathogen spreading and as a modulator of the signals and signaling pathways involved in the local and systemic response (Figure 1) (Cheval & Faulkner, 2018; Gupta et al., 2020; Houston et al., 2016; Kumar & Dasgupta, 2021; J. Liu et al., 2021; Nalam et al., 2019; Y. Wang et al., 2021). Deposition of callose in the outer cell wall is rarely seen in mutualistic symbiosis, suggesting that there are mechanisms to suppress this response when beneficial for the plant (Figure 1b). Microbes can enter plant cells via wounds or natural openings or directly injected by carriers (such as aphids). Fungi form a specialized cell (appressorium) to force their way into the outer cell layer, becoming later a feeding structure called hyphae that invade neighboring cells. Plants have evolved mechanisms to detect pathogen molecular patterns (a pathway named pattern‐triggered immunity) and react by reinforcing the cell wall with callose, among other components (Figure 1a). Adapted, virulent pathogens deliver effectors that suppress pattern‐triggered immunity, rendering plants defenseless against infection. Resistant hosts can activate effector‐triggered immunity, which often results in cell death (i.e., hypersensitive response). Downstream of pattern and effector‐triggered immunity, salicylic acid, jasmonic acid and ethylene‐mediated signaling pathways are activated, leading to the priming of defenses and systemic acquired resistance (W. Zhang et al., 2018). Biotrophic pathogens induce salicylic acid, which activates *NONEXPRESSOR OF PATHOGENESIS‐RELATED GENES 1*, an interactor of TGACG‐binding transcription factors in the nucleus, which regulates the expression of salicylic acid‐responsive genes encoding pathogenesis‐related proteins. Salicylic acid can be converted into methyl salicylate, which acts as a long‐distance signal to induce transcriptional reprogramming of defense genes in distant tissues/organs (Fu & Dong, 2013). The induction of jasmonic acid/ethylene‐mediated signaling pathways is associated with necrotrophic pathogens leading to the expression of defensins and other antimicrobial genes.

**Figure 3:**
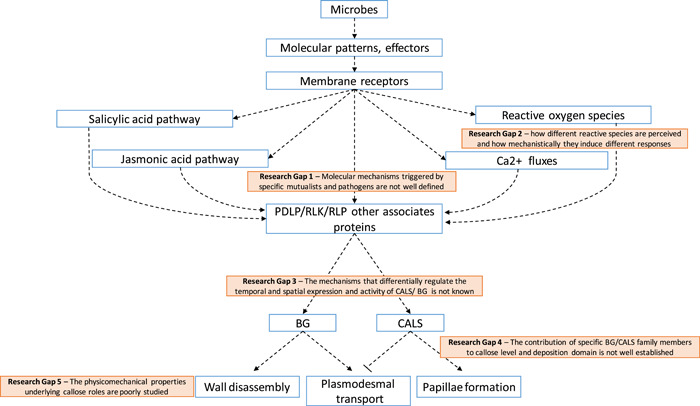
A schematic model highlighting research gaps in the understanding of the molecular mechanisms that regulate callose metabolism in response to microbial infection. Molecular patterns and effectors produced during microbial attack are perceived by plant membrane receptors inducing signaling pathways mediated by either salicylic acid, jasmonic acid, calcium fluxes and/or reactive oxygen species. The specific signaling pathway is highly dependent on the microbe−host plant interaction and, so far, it is not clear what distinguishes the mechanisms induced by mutualistic versus pathogenic microbes (Research Gap 1). Distinct signaling and responses might also depend on the type and levels of reactive oxygen species produced in the apoplast and inside the cell, but this mechanism is also poorly understood (Research Gap 2). The formation of homo‐ and heteromeric receptor complexes [among e.g., mitogen‐activated protein kinases (MAPKs), RLKs, RLPs, PDLPs] also determine these responses. These protein receptor complexes, and downstream signaling molecules modulate the activity of beta‐1,3 glucanases (BG) and callose synthases (CALS), which are responsible for callose turnover in cell walls. The activity of these enzymes can be controlled at the transcriptional and posttranscriptional level although the mechanisms remain unclear. CALS protein−protein interactions has been reported and modifications in the localization and abundance of these enzymes in the membrane (e.g., by changes in membrane composition or in the secretion pathway or their proteolysis) are also proposed as potential mechanisms (Research Gap 3). There is also a lack of knowledge of the differential contribution of members of the CALSs and BGs families to specific microbial responses which determine callose levels and localization (i.e., papillae or plasmodesmata) (Research Gap 4). It is suggested that callose synthesis reinforces cell walls against pathogen penetration (forming part of papillae) and restricts the transport of molecules via plasmodesmata whereas BG activity is part of the mechanism for cell wall disassembly (e.g., to generate damage associated molecular patterns) and contribute to systemic signaling by promoting plasmodesmata transport. It is yet unknown which physicomechanical properties support the function of callose in these different processes (Research Gap 5). For more information consult the text. PDLPs, plasmodesmata‐localized proteins.

The nuclear expression of callose metabolic genes is induced during pattern‐triggered immunity. In this process, the sensing of pathogen‐, microbe‐ or damage‐associated molecular patterns by plasma membrane‐localized receptors leads to the activation of calcium fluxes, production of extracellular reactive oxygen species by NADPH oxidases and the activation of MITOGEN‐ACTIVATED PROTEIN KINASES pathways that regulate gene expression (Nguyen et al., 2021) (Figure 3). A 22 aa peptide derived from bacterial flagellin (called flg22) and fungal wall chitin are examples of powerful pathogen‐associated molecular patterns. These are recognized by a family of membrane receptors (such as CHITIN‐ELICITED RECEPTOR KINASE 1 and FLAGELLIN SENSITIVE2) which in turn triggers reactive oxygen species and callose associated with pattern‐triggered immunity (Gómez‐Gómez et al., 1999; Jiménez‐Góngora et al., 2015). Callose is also induced posttreatment with lipopolysaccharides from *Liberibacter crescens* (a gram‐negative bacterium that infects *Nicotiana benthamiana*). Exposure to these pathogen‐associated molecular patterns induces a burst of nitric oxide and the expression of *NbCalS1* and *NbCalS12* (Jain et al., 2022). This effect can be reverted by treatment with the nitric oxide scavenger, 2‐(4‐carboxyphenyl)‐4,4,5,5‐tetramethylimidazoline‐1‐oxyl‐3‐oxide (cPTIO), suggesting that signaling pathways leading to callose deposition are dependent on the nitric oxide burst.

Studies using mutants in callose, specifically by targeting *GSL5* (mutant named *pmr4*) demonstrate the importance of callose during pattern‐triggered immunity. *pmr4* plants are impaired in response to infection with the bacteria *Pseudomonas syringae*, whereas overexpression of GSL5 restricts penetration of the powdery mildew fungal pathogen (Ellinger et al., 2013; A.K. Jacobs et al., 2003; Kim et al., 2005). High resolution imaging of callose‐cellulose network suggests that callose permeates cellulose microfibrils at the site of fungal penetration which reinforces cell walls (Eggert et al., 2014). This is supported by our studies reporting callose‐cellulose interactions using ionic liquid and hydrogels models (Abou‐Saleh et al., 2018). Although not yet verified in cell walls, mechanical determinations suggest that cellulose‐callose mixtures are more elastic (lower Young's modulus) and more ductile (higher yield point) than cellulose alone. GSL5/PMR4 overexpression does not over accumulate callose unless infected with the fungi suggesting that this enzymatic activity is posttranscriptionally regulated by pathogen‐induced factors. A follow‐up study identified RabA4c GTPase as one of the proteins involved in the activation and translocation of PMR4 to the plasma membrane, controlling its function and callose metabolism in response to infection with virulent powdery mildew (Ellinger & Voigt, 2014).

Parallelisms between pattern and effector‐triggered immunity in relation to callose regulation emerge from the literature. As described for pattern‐triggered immunity, callose deposition was induced when *Arabidopsis* plants were transformed with an inducible bacterial effector originating from *P. syringae* pv. pisi (AvrRps4) (Halane et al., 2018; Ngou et al., 2021). Crosstalk between the signaling pathways triggered by extracellular and intracellular receptors (coactivation of pattern and effector‐triggered immunity) results in stronger defenses and callose deposition than either immune system alone (Ngou et al., 2021). Effector proteins secreted by aphids also regulate BGs and CALSs (Silva‐Sanzana et al., 2020). Expression in *Arabidopsis* of the salivary protein MP55 from *Myzus persicae* suppresses callose accumulation (by up‐regulation of BGs), among other defenses, in response to aphid feeding (Elzinga et al., 2014). Overexpression in wheat of two specific bacterial effector proteins GroEL (Chaudhary et al., 2014) and GroES (Q. Li et al., 2022), secreted by the wheat aphid, *Sitobion miscanthi*, and produced by its bacterial endosymbiont *Buchnera aphidicola*, trigger the accumulation of reactive oxygen species, up‐regulation in the expression of three wheat GSL genes, callose deposition and a significant decrease in aphid fecundity. This suggests that plants have evolved defense strategies against aphids by recognizing effector proteins from their endosymbiotic bacteria leading to effector‐triggered immunity (Q. Li et al., 2022).

Besides regulating callose accumulation in plant cell walls, extracellularly released BGs can act as antimicrobial enzymes degrading fungal (and certain bacterial) cell walls. Enzymatic degradation of fungal beta‐1,3 glucan structures releases fragments or damage‐associated molecular patterns that activate the plant defense response (Kauffmann et al., 1987). These enzymes have been identified in many plant species, and their ectopic expression enhances the resistance against phytopathogenic fungi (Amian et al., 2011; Mackintosh et al., 2007; Wróbel‐Kwiatkowska et al., 2004).

Together, the examples discussed above highlight the importance of callose metabolism in pattern‐ and effector‐triggered immunity and uncover some of the signaling factors involved in the transcriptional and posttranscriptional regulation of callose metabolic enzymes. Components of the signaling pathways leading to callose regulation remain unidentified as well as how these different pathways integrate to specifically target cell wall reinforcement against pathogens and/or to trigger changes in cell‐to‐cell communication. Moreover, questions remain on which specific members of the BG family of enzymes acts in defense by degrading certain fungal and bacterial cell walls and how are they differentially regulated in relation to those acting at plasmodesmata to regulate intercellular transport and systemic responses.

While there is a multitude of evidence that links the regulation of callose and the plant response to the invasion of pathogens, very few examples evidence its role in mutualistic symbiosis. One of these examples emerged through the study of the plant protein VAPYRIN, which is essential for the establishment of symbiosis between leguminous roots with both arbuscular mycorrhizal fungi and rhizobia (Bapaume et al., 2019; M. Chen et al., 2021; C.W. Liu et al., 2019). *Petunia hybrida vapyrin* mutants infected with arbuscular mycorrhizal fungi form callose‐rich papillae around the penetrating fungal hyphae and surrounding the internal hyphae, preventing the symbiotic association (M. Chen et al., 2021). Similarly, mutations in *Pisum sativum L*. symbiotic genes *sym33* and *sym42* (impaired in rhizobia colonization) displayed an increase in callose deposition around infection threads (cytoplasmic invaginations that carry the bacteria into deeper tissues) and, occasionally, around the nodules (Ivanova et al., 2015; Tsyganova et al., 2019). Along with callose deposition, the mutants *sym33* and *sym42* exhibited thicker cell walls, depositions of unesterified pectins, and altered expression of pathogenesis‐responsive genes suggesting a general role for these proteins in silencing defense responses that can compromise symbiont colonization (Ivanova et al., 2015). These examples suggest that host plants modify cell walls and restrict callose deposition as a mechanism to promote beneficial symbioses, a process that appears dependent on the expression of *VAPYRIN, SYM33* and *SYM42* genes (Figure 1b).

Callose regulation is also linked to plant responses to certain exosymbiotic and endosymbiotic non‐pathogenic bacteria, also called plant growth‐promoting bacteria, and to growth‐promoting endophytic symbiotic fungi (e.g., *Piriformospora indica*) (S. Jacobs et al., 2011; Jogawat et al., 2020). Induction of callose in these interactions play a dual role in strengthening plants defenses against potential pathogens and in restricting the non‐pathogenic infection to levels that are not prejudicial to plant growth. Callose was induced in rice roots treated with either the growth promoting bacterium *Bacillus subtilis* or the flavonoid Rutin or with a combination of both treatments (A. Singh et al., 2016). Significant cell wall thickening and callose depositions were also seen in strawberry leaves 72 h after infection with the plant growth‐promoting bacteria, *Azospirillum brasilense* (Guerrero‐Molina et al., 2015). The systemic disease resistance conferred by plant growth‐promoting bacteria and the subsequent callose priming have also been tested in tandem with arbuscular mycorrhizal fungi. The overall callose deposition was five times higher in leaves of wheat plants treated with both *Rhizophagus irregularis* (arbuscular mycorrhizal fungi) and *Pseudomonas putida* KT2440 (a plant growth‐promoting bacteria) than when treated with any of these microbes alone (Pérez‐De‐Luque et al., 2017). This multiplicity effect was seen in the wheat variety Mercato but not in Avalon, suggesting differential regulation of callose metabolism in the different genotypes.

The mechanism regulating callose levels in response to mutualistic symbiosis has not been fully dissected. Jogawat et al. (2020) showed that when infected with the fungi *P. indica*, *Arabidopsis* plants mutated in the Ca^2+^ channel CYCLIC NUCLEOTIDE GATED CHANNEL 19 (CNGC19) showed a delay in callose deposition in comparison to wild type. *cngc19* plants displayed a significant increase in fungal colonization to the detriment of plant growth suggesting that the channel activity is vital for maintaining a purely mutualistic symbiosis between *P. indica* and *Arabidopsis*. Interestingly, pattern‐triggered immunity responses were also compromised in *cngc19* leading to pathogenic invasions.

To summarize, the initial regulation of callose metabolism is a general response to pathogenic attack and it is associated with the activation of pattern‐ and effector‐triggered immunity. Callose synthesis mechanically reinforces cell walls by interacting with cellulose microfibrils which is important to restrict pathogen invasion. Some BGs are secreted to digest fungal and bacterial cell walls releasing damage‐associated molecular patterns that trigger defense responses. Mutualistic microbes have evolved different strategies to evade plant defenses, some including down‐regulation of the pathways that induce callose. Plants have also adapted by differentially activating genes that regulate callose in response to mutualistic or parasitic symbionts. CALS and BG genes and other key regulatory proteins and factors have been identified to control callose in response to microbial invasions, but we lack a complete mechanistic understanding of these processes. We also do not know how the mechanisms for cell wall reinforcement interact with those that regulate callose to control intercellular transport via plasmodesmata tightly linked to local and systemic responses, a topic that we discuss in the next section.

## 4 THE ROLE OF PLASMODESMATA‐ASSOCIATED CALLOSE IN PLANT SYMBIOTIC INTERACTIONS

Plasmodesmata‐manipulation by, and in response to, diverse microbes have been recently reviewed by other authors (Figure 1) (Dorokhov et al., 2019; Ganusova & Burch‐Smith, 2019; Huang & Heinlein, 2022; J. Liu et al., 2021; Y. Wang et al., 2021). The best‐characterized mechanism is in response to viruses. Infection with Tobacco mosaic virus (TMV) or the Potato virus X initiates early callose deposition at plasmodesmata which restricts virus spreading. These viruses encode a viral movement protein that interacts with plasmodesmata components (including BGs) and modify callose to facilitate their spreading (Huang & Heinlein, 2022). The activation of callose in response to virus infection is crucial as a mutation of 8 amino acid residues within the HELPER COMPONENT PROTEINASE enables a virulent mutant strain of the Potato virus Y to evade the pattern‐triggered immunity response and the associated callose deposition (Chowdhury et al., 2020).

Studies on the molecular mechanism underlying the regulation of callose during virus infection identified REMORIN1.3 (a plant protein that localizes at lipid rafts and plasmodesmata) and a response dependent on the abscisic acid and salicylic acid signaling pathways. Ectopic expression of REMORIN1.3 in tobacco increases plasmodesmata‐callose and affects virus infection, although this response varies such that the spreading of tobamoviruses (e.g., TMV) are restricted, but potyviruses (e.g., Turnip mosaic virus or Potato virus A) are enhanced (Rocher et al., 2022). The potyviral movement protein cylindrical inclusion was found to interact with REMORIN1.3 counteracting its role in promoting callose at plasmodesmata. Induction of abscisic acid and salicylic acid signaling mechanisms also restricts virus spreading. In a recent study, tobacco plants were treated with polypeptide extracts from the dry mycelium of *Penicillum chrysogenum*, which activates an abscisic acid‐dependent mechanism that reduces BG expression (Y. Li,  et al., 2021). This treatment primes the plant defenses against TMV; thus, when exposed to the virus, callose over‐accumulates at the cell wall near plasmodesmata leading to a reduction in plasmodesmata diameter by around 10 nm. An increase in abscisic acid levels (exogenous or endogenous) was correlated more generally with increased callose deposition and a defense response to several types of pathogens in various species such as tobacco necrosis virus in *Phaseolus vulgaris*, bamboo mosaic virus in *Arabidopsis*, and *Nilaparvata lugens* in rice (Alazem & Lin, 2017; Iriti and Faoro, 2008; J. Liu et al., 2017). Similarly, TMV spreading was restricted by a salicylic acid‐triggered response in *N. benthamiana* leaves infiltrated with an extracellular subtilase isolated from the fungal pathogen, *Acremonium strictumelicitor* or treated with a biostimulant named Plant Stimulation and Protection 1 (Caro et al., 2022; Chalfoun et al., 2018). A transgenic line with reduced salicylic acid, due to misexpression of the bacterial salicylic acid hydroxylase gene *NahG* (Gaffney et al., 1993), failed to induce callose in response to *A. strictumelicitor* treatment failing to restrict TMV (Caro et al., 2022; Tsuda et al., 2008).

The induction of callose at plasmodesmata mediated by salicylic acid‐dependent mechanisms also play a role in defense against bacterial pathogens. Callose is deposited in response to infection with the virulent bacteria *P. syringae pv maculicola* by a mechanism that involves induction of the plasmodesmata receptor protein PDLP5 in a salicylic acid‐dependent pathway (J.Y. Lee et al., 2011). Ectopic expression of PDLP5 activates callose via the expression of CALS1 and CALS8 (Cui & Lee, 2016). In the PDLP5 overexpression background, ectopic expression of *NahG* or mutations in a known regulator of salicylic acid signaling: *NONEXPRESSOR OF PATHOGENESIS‐RELATED GENES 1*, led to reduced callose deposition and enhanced symplasmic transport (X. Wang et al., 2013). This finding indicates the importance of salicylic acid signaling in the PDLP5 mediated callose‐regulatory mechanism.

Effector triggered immunity also regulate callose at plasmodesmata by a mechanism involving PDLPs (Z. Li, et al., 2021). An effector protein secreted by *P. syringae* interacts and degrades PDLP5 and PDLP7, altering callose deposition and promoting plasmodesmata transport (Aung et al., 2020). Another effector protein secreted by the oomycete *Phytophthora brassicae* overrides PDLPs and can directly target plasmodesmata and interact with CALS to regulate callose and intercellular transport (Tomczynska et al., 2020). As described in the previous section, flg22 induces callose deposition and this bacterial pattern also restricts plasmodesmata transport. An *Arabidopsis* CALMODULIN‐LIKE protein 41 (CML41), which localizes at plasmodesmata and binds calcium, was induced post flg22 treatment. CML41‐amiRNA lines showed no induction in plasmodesmata callose upon treatment with flg22, whereas a CML41 overexpression line displays higher callose. Callose induction was reduced by a calcium chelator suggesting that calcium signaling is important in this response (B. Xu et al., 2017). Besides calcium, reactive oxygen species also accumulate in response to flg22 and a mutant in the NADPH oxidase RBOHD, which fails to induce hydrogen peroxide in response to flg22 treatment, was found impaired in the callose response (Luna et al., 2011). Interestingly, a recent report indicates that RBOHD‐ activity in the regulation of cell‐to‐cell transport is dependent on PDLP1 and PDLP5 function (Fichman et al., 2021).

Besides bacteria and viruses, infection with other pathogens has been associated with callose regulation at plasmodesmata. When infecting *Arabidopsis*, the oomycete *Hyaloperonospora arabidopsidis* induces callose deposition around the invasive haustoria. This effect is not seen in the *pdlp1,2,3* triple mutant which appears more susceptible to the infection (Caillaud et al., 2014). Parasitic nematodes, including *Meloidogyne graminicola* and the cyst nematode *Heterodera schachtii*, also regulate callose altering plasmodesmata communication (reviewed by Rodiuc et al., 2014). Giant cells formed in rice plants infected with *M. graminicola* are symplasmically connected to the phloem according to dye unloading assays (L. Xu et al., 2021). Artificial manipulation of callose through *OsGNS5* (a BG) and *OsGSL2* (a CALS) affects the sugars found in the galls and nematode numbers, indicating that low callose levels are required for proper feeding and reproduction. In support, an effector protein from *M. graminicola*, MgMO237, was identified to suppress the host immune system and callose deposition (Chen et al., 2018). Previous work studying the syncytia (feeding structure) of the cyst nematode *H. schachtii* infecting *Arabidopsis* found reduced syncytia and reduced female: male ratio in a BG mutant impaired in the degradation of plasmodesmata‐callose (Hofmann et al., 2010).

While minimal, some research has been conducted on callose‐regulation at plasmodesmata in mutualistic symbioses (Figure 1b). In assays testing plasmodesmata connectivity in *M. truncatula* roots, Complainville et al. (2003) found that 5(6)‐carboxyfluorescein (a chemical that once modified in the cell becomes membrane impermeable thus, widely used as a reporter for symplasmic permeability), and free/cytoplasmic‐GFP can diffuse from the phloem initials into nodule primordia suggesting that these tissues are symplasmically connected. When connectivity was altered by ectopic expression of the TMV movement protein, the number of plasmodesmata increased, as well as the number of nodules. Although callose was not evaluated in this study, expression of TMV movement protein has been shown to affect callose degradation. A more recent study found a reduction in callose levels as early as 24 h after rhizobia infection of *M. truncatula* roots which correlate with an increase in the expression of an endogenous plasmodesmata‐located BG (MtBG2) (Gaudioso‐Pedraza et al., 2018). Ectopic MtBG2 expression improves nodulation, whereas silencing MtBG2 or ectopically expressing a hyperactive CALS3 (*cals3m*) under an infection‐specific promoter leads to defective infection and severe reduction in the number of nodules. PDLPs also seem to play a role in this mechanism as ectopic expression of a PDLP‐like protein identified in *M. truncatula*, alters callose and improves infection and nodulation in the presence of nitrate (Kirk et al., 2022).

To summarize, the examples discussed in this section indicate that the regulation of callose at plasmodesmata is a key component in the establishment of both parasitic and mutualistic symbiosis. Both pattern‐ and effector‐triggered immunity induce callose deposition at plasmodesmata via a mechanism involving salicylic acid signaling, calcium and reactive oxygen species leading to the activation of PDLPs and CALSs. Some viruses, nematodes and certain mutualistic symbionts have evolved to override this mechanism triggering activation of BGs. What is not clear yet is the temporal and spatial signaling mechanisms that differentially activate CALS or BG to determine symplasmic connectivity, intercellular molecular transport and different plant responses to beneficial or pathogenic microbial infection.

## 5 DISCUSSION

The plant cell wall represents the first line of defense against environmental stresses. The dynamic regulation of its structural components determines its physicomechanical properties and its capability to stop the penetration of parasitic microbes or engage in positive mutualistic symbiosis. In this review, we discussed the role callose plays in cell wall function, specifically focusing on the current understanding of the mechanisms that regulate its accumulation in response to microbes (Figure 1). The temporal and spatial regulation of callose deposition changes the properties of cell wall microdomains and determines the success of microbial infection. By accumulating callose at the site of infection, plants can block the entry of undesired microorganisms. This is linked to callose property to integrate with cellulose microfibrils changing its elastic properties and ductility. Changes in plasmodesmata‐callose are also triggered to promote the transport of signaling factors that alert and/or prepare neighboring tissues to respond to microbial attack. We discussed examples from the literature where callose‐mediated plasmodesmata regulation affects the spreading of microbes by modulating the cell‐to‐cell movement of viruses' movement proteins and pathogenic effectors. We also highlighted examples where callose degradation mediates the establishment of interactions with mutualistic symbionts whereas its synthesis restricts the infection of growth‐promoting bacteria and fungi to levels that are non‐pathogenic for the host plant. Clearly, plant−microbe coevolution has led to a diversification in the strategies to manipulate callose, but more work is required to identify the mechanisms that positively influence the establishment of beneficial interactions and deter pathogenic invasions.

Plants trigger different signaling mechanisms upon perception of beneficial and pathogenic microbes leading to differential callose regulation at and outside plasmodesmata microdomains. Multiple signaling molecules (salicylic acid, reactive oxygen species, Ca,^2+^ abscisic acid and so forth) and proteins (such as RBOHD, CML41, PDLPs) are involved in fine‐tuning the differential expression (or activity) of CALS and BG to dynamically control the properties of cell walls (Figure 3). Transcriptomic analysis carried out in symbiotic systems indicate that the expression of genes encoding for both, CALSs and BGs are regulated in response to parasitic and mutualistic infections (Dhokane et al., 2016; Gao et al., 2022; Golkari et al., 2007; Kirk et al., 2022; Marwein et al., 2022). Little is known about their posttranscriptional regulation, their interactions with other proteins and the spatial and temporal parameters affecting their function. As part of a multicomponent complex, CALS activity and targeting to plasmodesmata is controlled posttranscriptionally by changes in cytosolic calcium, phosphorylation and proteolysis (Schneider et al., 2016 and references therein). Interaction of CALS with membrane receptors such as PDLPs has also been hypothesized (De Storme & Geelen, 2014). On the other hand, membrane targeting of GPI‐anchored BGs can be affected by phospholipases and the sphingolipid/lipid raft composition of the plasmodesmata membranes (Iswanto et al., 2020). Research on proteins and posttranslational mechanisms affecting CALS and BG activity and membrane targeting could identify novel strategies to modify callose in response to specific microbes.

Another area where research is lacking concerns the reactive oxygen species and their specific involvement in determining plasmodesmata and callose regulation in both, mutualistic and parasitic interactions. Oxidative burst is described as one of the first response to pathogenic microbes, but this is also generated during rhizobia infection (Hu et al., 2021) and in arbuscular mycorrhizal symbiosis (Belmondo et al., 2016). Previous studies indicate that treatment with superoxide and nitric oxide induce callose whereas moderated levels of hydrogen peroxide improve symplasmic connectivity (Fichman et al., 2021). Moreover, the origin of these species, either by membrane NADPH oxidases or by intracellular organelles such as plastids or mitochondria, and the plant capacity to maintain cell redox homeostasis are also important factors. Large gaps in knowledge remain on how different reactive species are perceived inside the cell and how mechanistically they induce pattern triggered immunity, systemic acquired resistance or if they are involved in regulating plasmodesmata during mutualistic symbiosis (Figure 3).

Figure 3 highlights some of the challenges that we face in understanding the signaling molecules and membrane receptors that specifically target callose regulation in response to microbial attack (Figure 3 Research Gap 1 and 2). It also highlights other gaps in knowledge such as the nature of interactions between protein receptor complexes and BG/CALS and other posttranscriptional regulatory factors affecting the secretion and localization of these enzymes (Figure 3 Research Gap 3). Additionally, the regulation and contribution of different members of the CALS and BG families to cell wall reinforcement and/or to cell‐to‐cell communication is unknown (Figure 3 Research Gap 4). Furthermore, research is required to improve understanding of callose's physicomechanical properties and how this polysaccharide integrates with other cell wall components to either reinforce cell walls or to control transport via plasmodesmata (Figure 3 Research Gap 5).

In spite, opportunities have emerged to exploit interactions between callose regulatory pathways. For example, callose‐priming occurring after infection with mycorrhizal fungi and plant growth‐promoting bacteria has been shown to enhance plant resistance to infections with microbial pathogens. This was seen after infection of tomato roots with the arbuscular mycorrhizal fungi *Glomus mosseae*, which enhanced callose deposition in cell walls surrounding the intercellular hyphae of the pathogenic oomycete *Phytophthora parasitica* (Cordier et al., 1998). Similarly, Sanmartín et al. (2020) showed that tomato plants inoculated with *R. irregularis* induced higher levels of callose and displayed smaller mycelium diameters during infection with the necrotic fungus *Botrytis cinerea*. More research to identify the factors involved in callose regulation during these microbial interactions would be instrumental in the design of biotechnological approaches to target callose as a tool for crop improvement. When modifying callose we must also take into consideration the potential side effects on plant growth as callose at plasmodesmata regulate the expression of developmental signals, transcription factors, RNAs and metabolites. The newly described role of plasmodesmata in the establishment of an auxin gradient (Band, 2021) also opens questions on targeting callose, as auxin affects both infection and formation of lateral organs and feeding structures such as nitrogen‐fixing nodules, nematode‐induced giant cells, or arbuscular structures during arbuscular mycorrhizal symbiosis.

To conclude, a mechanistic understanding of the molecular components underlying the regulation of callose during papillae formation or that control transport via plasmodesmata is still lacking (Y. Wang et al., 2021). Questions also remain on the importance of callose in regulating other processes such as molecular diffusion via the cell wall (apoplastic transport), cell wall hydration, porosity, adhesion and elasticity and how these physicomechanical properties influence its role in symplasmic transport and defense. Knowledge is missing on the factors that enable pathogen resistance without affecting beneficial symbioses. Better understanding of the mechanisms controlling these responses offers new opportunities to exploit the differential regulation of callose in sustainable agriculture.

## AUTHOR CONTRIBUTIONS

All authors were involved in the review writing. Richa Yeshvekar and Liam German generated the first draft and designed the figures. Yoselin Benitez‐Alfonso designed the review, was involved in the manuscript writing and reviewing, integrated comments and contributions from the different authors and reviewers and generated the final submission.

## CONFLICT OF INTEREST

The authors declare no conflict of interest.

## Data Availability

Data sharing is not applicable to this article as no new data were created or analyzed in this study.
